# From Screening to Delivery of Disease‐Modifying Therapy: Real World Follow‐Up of Children With Early‐Stage Type 1 Diabetes

**DOI:** 10.1111/dom.70689

**Published:** 2026-03-30

**Authors:** Theodora Papanikolaou, Josephine Elliott, Veer Sheth, Lauren M. Quinn, Parth Narendran, Renuka P. Dias

**Affiliations:** ^1^ Institute of Immunology and Immunotherapy University of Birmingham Birmingham UK; ^2^ Institute of Cancer and Genomic Sciences University of Birmingham Birmingham UK; ^3^ Birmingham Medical School University of Birmingham Birmingham UK; ^4^ Department of Paediatric Endocrinology, Birmingham Children's Hospital Birmingham UK; ^5^ Institute of Applied Health Research University of Birmingham Birmingham UK

**Keywords:** autoimmunity, disease prevention, real‐world evidence, type 1 diabetes

## 1 Background

Paediatric Type 1 diabetes (T1D) screening studies, including the UK‐based EarLy Surveillance for Autoimmune Diabetes (ELSA) study [1], have enabled wider identification of early‐stage T1D in children, defined by the presence of two or more islet autoantibodies (IAb) and associated glycaemic patterns [2].

However, screening alone cannot prevent diabetic ketoacidosis (DKA) or optimise long‐term glycaemic outcomes [3, 4]; structured surveillance is essential, and qualitative research indicates that families value ongoing support and clinical guidance following an early‐stage diagnosis [5].

To support the ongoing need to monitor children with screen‐detected early‐stage T1D from ELSA, we established a national early‐stage T1D clinic at Birmingham Children's Hospital in April 2024.

Here, we report our early lessons from delivering structured follow‐up to children across England during a period of rapidly expanding early detection.

## 2 Methods

A retrospective case‐note review was undertaken for all children attending the early‐stage T1D clinic between April 2024 and December 2025. Children under 16 years across the UK were eligible if they had early‐stage T1D, defined as ≥ 2 IAb and no requirement for insulin therapy (Table S1). A parental experience survey was completed as part of routine service evaluation.

### 2.1 Clinic Structure

The clinic uses a hybrid model, offering in‐person and virtual consultations within a shared‐care framework with local paediatric diabetes teams and their general practitioners. It is led by an experienced paediatric endocrinologist with expertise in early‐stage T1D. Follow‐up frequency was determined by age and stage and aligned with international consensus guidance (Figure S1) [6].

Glycaemic monitoring included venous glycated haemoglobin (HbA1c), intermittent self‐monitored blood glucose (SMBG) and periodic continuous glucose monitoring (CGM), depending on local team capacity and family preference (Figure S1). Oral glucose tolerance tests (OGTTs) were undertaken through INNODIA [7] research sites or local hospitals. All patients had access to a capillary blood glucose (CBG) meter provided by their general practitioner (GP) or local diabetes team, with training arranged locally. Families were advised to check blood glucose during episodes of osmotic symptoms or illness, and to perform SMBG 2 h post carbohydrate‐rich meal monthly if not using CGM [6, 8].

Eligible patients were assessed for disease‐modifying therapy (DMT). Psychological support was available as part of a wraparound care model.

## 3 Statistics

Descriptive data are presented as medians with interquartile ranges (IQR).

## 4 Results

**FIGURE 1:**
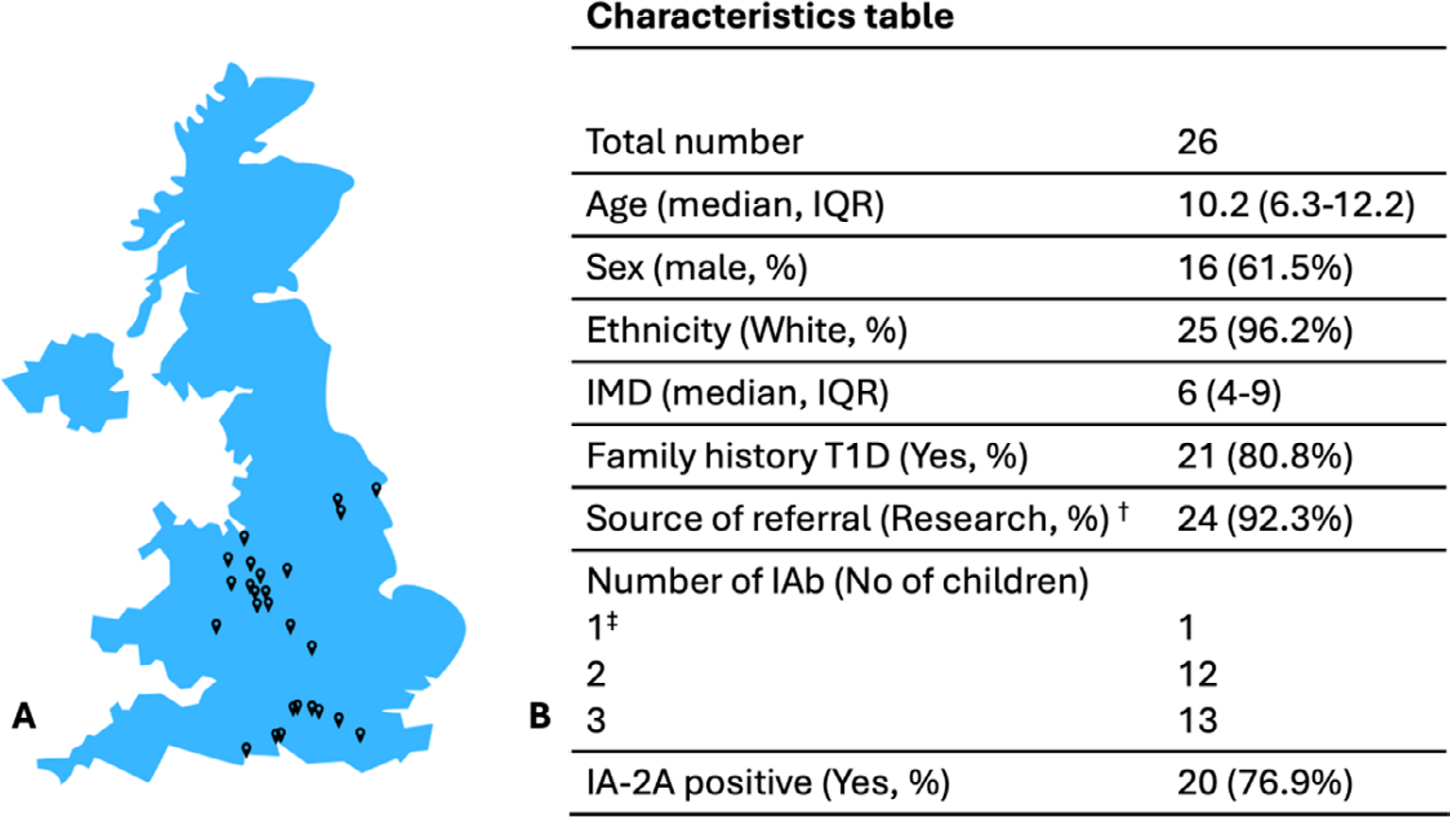
Geographic distribution of referred children and baseline cohort characteristics. (A) Map of England showing the geographical distribution of the 26 children referred to the early‐stage Type 1 diabetes clinic in Birmingham. (B) Baseline characteristics of the clinic cohort. Values shown as medians with interquartile ranges or counts with percentages, as appropriate. † Referrals through clinical care comprised one child identified during routine care at Stage 3a, for whom insulin initiation support was provided, and one child initially identified through research who subsequently underwent confirmatory testing and staging via standard clinical care following clinical concerns. ‡ One child had single islet autoantibody positivity confirmed across two independent laboratories (research and clinical) and was classified as Stage 2 based on dysglycaemia criteria; therefore, accepted for follow‐up in the clinic. IQR, interquartile range; IMD, index of multiple deprivation; T1D, type 1 diabetes; IAb, islet autoantibody; No, number; IA‐2A, insulinoma‐associated‐2 autoantibody.

Twenty‐six children were followed during the 21‐month period. Twenty‐four (92.3%) were referred from research (ELSA) [1] and two from clinical care. Most patients were from the West Midlands (*n* = 10). However, the geographical spread of the patients was wide, covering different regions across England (Figure 1).

The median age at detection of IAb positivity was 10.2 years (IQR 6.3–12.2). Sixteen children were male. Twenty‐one (80.8%) had a family history of T1D, and all but one child were of White ethnicity. Socioeconomic diversity was broad, with a median index of multiple deprivation (IMD) decile of 6 (IQR 4–9).

At referral, 16 children were in Stage 1, nine in Stage 2, and one in Stage 3a.

Nineteen children underwent at least one repeat OGTT (15 via INNODIA [7]; 4 via clinical care). The remaining seven were monitored using HbA1c and intermittent SMBG; five additionally used intermittent CGM.

**FIGURE 2:**
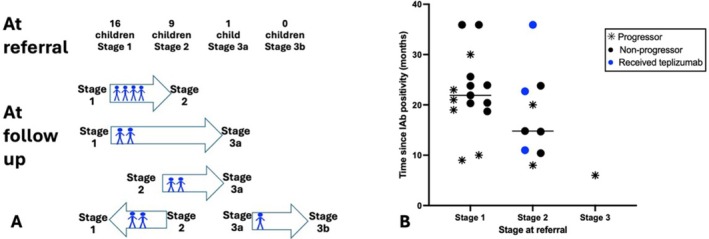
Clinical stage transitions and follow‐up time since islet autoantibody positivity. (A) Flow diagram showing stage at referral and subsequent stage at follow‐up. During follow‐up, nine children progressed and two reverted between stages. The remaining children remained in the same stage during the follow‐up period. (B) Time (in months) since islet autoantibody (IAb) positivity detected for progressors and non‐progressors, stratified by stage at referral. Each point represents one participant. * Progressors: Time from IAb positivity to confirmed stage progression. Non‐progressors: Duration of follow‐up since IAb positivity detected. Children who received teplizumab: Duration of follow‐up since IAb positivity detected. Horizontal lines indicate group medians. One child referred in Stage 1 was discharged to local care and is therefore not included in Panel B. IAb = islet autoantibody.

During follow‐up, four children (25.0%) in Stage 1 progressed to Stage 2, two (22.2%) of those in Stage 2 progressed to Stage 3 and two (12.5%) from Stage 1 to Stage 3. No child who progressed to Stage 3 presented with DKA. Conversely, 22.2% (2/9) of Stage 2 children reverted to Stage 1. Median time from detection of IAb positivity to stage progression among progressors was 19.5 months (IQR 9.3–22.5). Among non‐progressors, the median time since IAb positivity at the time of analysis was 22.3 months (IQR 15.8–25.2, range 10.4–35.9) (Figure 2). No statistically significant differences in age, antibody number, IA‐2A titres, 90‐min OGTT values or duration of follow up were observed between progressors and non‐progressors, although small numbers limit interpretation.

In five of the six progressors, HbA1c alone would have identified stage change (> 10% rise from previous visit) [6, 8]. One child had only a 9% HbA1c increase at first follow‐up and progression to Stage 2 would not have been detected without OGTT.

Eight children met eligibility criteria for disease‐modifying therapy (teplizumab) through a managed access programme. Three received treatment without significant adverse events, and one was awaiting treatment at analysis. Expected mild side effects (including transient lymphopenia and elevation of liver enzymes, rash) were observed but none required treatment interruption or discontinuation. Two families chose not to proceed, primarily due to travel burden. Two eligible children progressed to Stage 3 before treatment could be delivered.

No family required referral for psychological support. Eight survey responses were received from seven families; feedback was uniformly positive with all respondents rating the clinic as useful or very useful, and most reported increased confidence and understanding following review (Figure S2).

## 5 Conclusion

Our early experience in a small, evolving cohort highlights three key themes:

1. *Children can progress between stages quickly—and movement is not always linear*. Regular surveillance enabled early identification of progression before symptoms emerged and, crucially, none of the four children who progressed to Stage 3 presented in DKA. In keeping with BSPED guidance [8], HbA1c with intermittent SMBG would have detected progression in most cases; however, one child would not have met criteria for stage change without an OGTT. While HbA1c‐based monitoring is appropriate for routine clinical follow‐up, selective OGTT use with individualised risk stratification, including lower HbA1c thresholds, may support timely identification when disease‐modifying therapy is considered.
  Reversion from Stage 2 to Stage 1 was observed, consistent with longitudinal cohort data showing that dysglycaemia may transiently normalise, supporting a dynamic and variable rather than strictly linear disease trajectory [9]. Such fluctuations may reflect intercurrent illness, measurement variability, or variation in insulin sensitivity during the preclinical phase [10]. Conversely, two children eligible for teplizumab progressed rapidly to Stage 3 before treatment could be delivered, highlighting a narrow therapeutic window in some individuals. Together, these observations emphasise heterogeneity among individuals within the same stage and suggest that incorporation of emerging biomarkers may improve individual risk stratification beyond current staging frameworks [11].
2. *Ongoing follow‐up is welcomed by families*. Parents rated the clinic positively and no family required psychology referral, suggesting the model may help manage the uncertainty of an early‐stage diagnosis. Interest in teplizumab was high among eligible families (6/8). As real‐world efficacy data emerge, pragmatic monitoring approaches may support timely identification of individuals eligible for disease‐modifying therapy [12].
3. *Equity of access must be prioritised*. Our cohort differed from typical UK paediatric T1D clinics (80.8% with family history of T1D, 96.2% White ethnicity), compared with ~15% and 82% respectively in the broader clinic population [13]. This likely reflects the referral pathway: most children were identified through the ELSA study, where 71.1% of antibody‐positive participants had a family history [1]. Although ELSA recruits an ethnically representative cohort (18.3% non‐White, comparable to 20.9% nationally) [1, 14]. White children were more likely to have multiple autoantibodies (1.05% vs. 0.42% in minority ethnic groups), a difference attenuated after adjusting for family history [1]. These patterns may partly explain the over‐representation of children of White ethnicity in our clinic, though other factors may also contribute. Socioeconomic representation was more balanced, but targeted strategies remain essential to ensure equitable access, particularly as underserved groups face higher risks of DKA at diagnosis and poorer long‐term outcomes [15].

This is the first description of a UK cohort of children with early‐stage T1D managed in a real‐world clinical setting. Our early experience suggests that a hybrid shared‐care model is feasible and clinically valuable, providing regular surveillance, timely recognition of progression, and family support while minimising burden. Without this, the benefits of early detection may not be fully realised. Embedding early‐stage T1D management into routine care ensures families are not left navigating a presymptomatic diagnosis alone and lays the foundation for improved long‐term outcomes. Looking ahead, national initiatives—including training resources for healthcare professionals and the recent appointment of early‐stage T1D clinical champions across the UK—could facilitate wider implementation, enabling scalable delivery while maintaining specialist oversight.

## Author Contributions

T.P. and R.P.D. researched data and wrote the first draft of the manuscript. J.E. and V.S. devised the patient experience survey. V.S. analysed the anonymous patient experience data. J.E., V.S., L.M.Q. and P.N. reviewed and edited the final manuscript. All authors approved the final version of the manuscript. R.P.D. is the guarantor of this work and, as such, had full access to all the data in the study and takes responsibility for the integrity of the data and the accuracy of the data analysis.

## Funding

No funding has been received for this work. R.P.D. is supported by National Institute for Health Research Award (Ref NIHR304587). J.E. is supported by National Institute for Health Research Award (Ref ACF‐2023‐09‐004).

## Ethics Statement

This study was conducted as part of a service evaluation and registered with the institutional audit department (Audit Registration Number: CARMS‐32012).

## Consent

All data were collected as part of routine clinical care. In accordance with local and national guidelines for service evaluations, formal ethical approval was not required, and no patient consent was necessary.

## Conflicts of Interest

R.P.D. has received honoraria from Sanofi for participation in an advisory board on teplizumab in Type 1 diabetes and speaker fees from Sanofi and Sandoz. T.P. received honoraria from Sanofi (participation in an advisory workshop for teplizumab in Type 1 diabetes and speaker fees). P.N. was a clinical expert for the National Institute for Clinical Excellence (NICE) on the development of teplizumab in January 2024 and has received an honorarium from Sanofi for participating on an advisory board on teplizumab and for speaking at national meetings on screening for Type 1 diabetes. Other authors (J.E., V.S., L.M.Q.) have no conflicts of interest to declare.

## Supporting information


**Table S1:** Autoantibody assessment and clinical framework for referral, staging and treatment eligibility.
**Figure S1:** Surveillance strategy used in the early‐stage Type 1 diabetes clinic.
**Figure S2:** Family survey responses before and after clinic attendance.

## Data Availability

The data that support the findings of this research letter are available from the corresponding author upon reasonable request.
