# Supplementary material for: From Screening to Delivery of Disease‐Modifying Therapy: Real World Follow‐Up of Children With Early‐Stage Type 1 Diabetes
Source: Diabetes Obes Metab. 2026 Mar 30;28(6):5379–83. doi: 10.1111/dom.70689 (PMC13146209; doi:10.1111/dom.70689)
Supplement: Supplementary file 1 — Table S1: Autoantibody assessment and clinical framework for referral, staging and treatment eligibility. Figure S1: Surveillance strategy used in the early‐stage Type 1 diabetes clinic. Figure S2: Family survey responses before and after clinic attendance. [file DOM-28-5379-s001.docx]

**Supplementary materials**

**Supplementary table 1.** Autoantibody assessment and clinical framework for referral, staging and treatment eligibility.

| **Domain** | **Details** |
| --- | --- |
| Islet autoantibodies measured | GAD  IA-2A  ZnT8  IAA |
| Eligibility for referral to clinic | Children and young people less than 16 years of age and  ≥2 islet autoantibodies present ^†^ and  No insulin requirement |
| Referral source | - Research study (ie ELSA study) - Clinical care |
| Stage 1 T1D | ≥2 autoantibodies with normoglycaemia   - FPG <5.6 mmol/L and - 2-h PG <7.8 mmol/L and - HbA1c <39 mmol/mol |
| Stage 2 T1D | ≥2 autoantibodies with dysglycaemia:   - FPG 5.6–6.9 mmol/L or - 2-h PG 7.8–11.1 mmol/L or - intermediate OGTT glucose at time points (30, 60, 90 min) ≥11.1 mmol/L or - HbA1c 39-47 mmol/mol or ≥10% increase from the first measurement with stage 2 |
| Stage 3 T1D   - Stage 3a asymptomatic - Stage 3b symptomatic | Fulfilling ADA criteria:^2^   - FPG ≥7.0 mmol/L or - 2-h PG ≥11.1 mmol/L or - HbA1c ≥ 48 mmol/mol   Diagnosis of Stage 3 T1D in the absence of symptoms required confirmatory testing. |
| Teplizumab eligibility | Meeting two stage 2 criteria at the same time point or one criterion on two separate time points. |

**Supplementary Table 1. Legend:** Islet autoantibodies were measured using validated assays within research screening programmes or local clinical laboratories. Stage classification followed ISPAD-based criteria.^1^ Dysglycaemia for Stage 2 classification was defined by the presence of at least one abnormal glycaemic criterion. Stage 3 diagnosis followed ADA diagnostic criteria and required confirmatory testing in asymptomatic individuals.

† At least 2 samples tested at different time points with different validated assays.

**Abbreviations:** GAD = glutamic acid decarboxylase autoantibody; IA-2A = insulinoma-associated-2 autoantibody; ZnT8 = zinc transporter 8 autoantibody; IAA = insulin autoantibody; ELSA = EarLy Surveillance for Autoimmune diabetes study; T1D = type 1 diabetes; OGTT = oral glucose tolerance test; HbA1c = glycated haemoglobin; FPG = fasting plasma glucose; PG = plasma glucose; ADA = American Diabetes Association; ISPAD = International Society for Pediatric and Adolescent Diabetes.

**Supplementary figure 1** Surveillance strategy used in the early-stage type 1 diabetes clinic

**
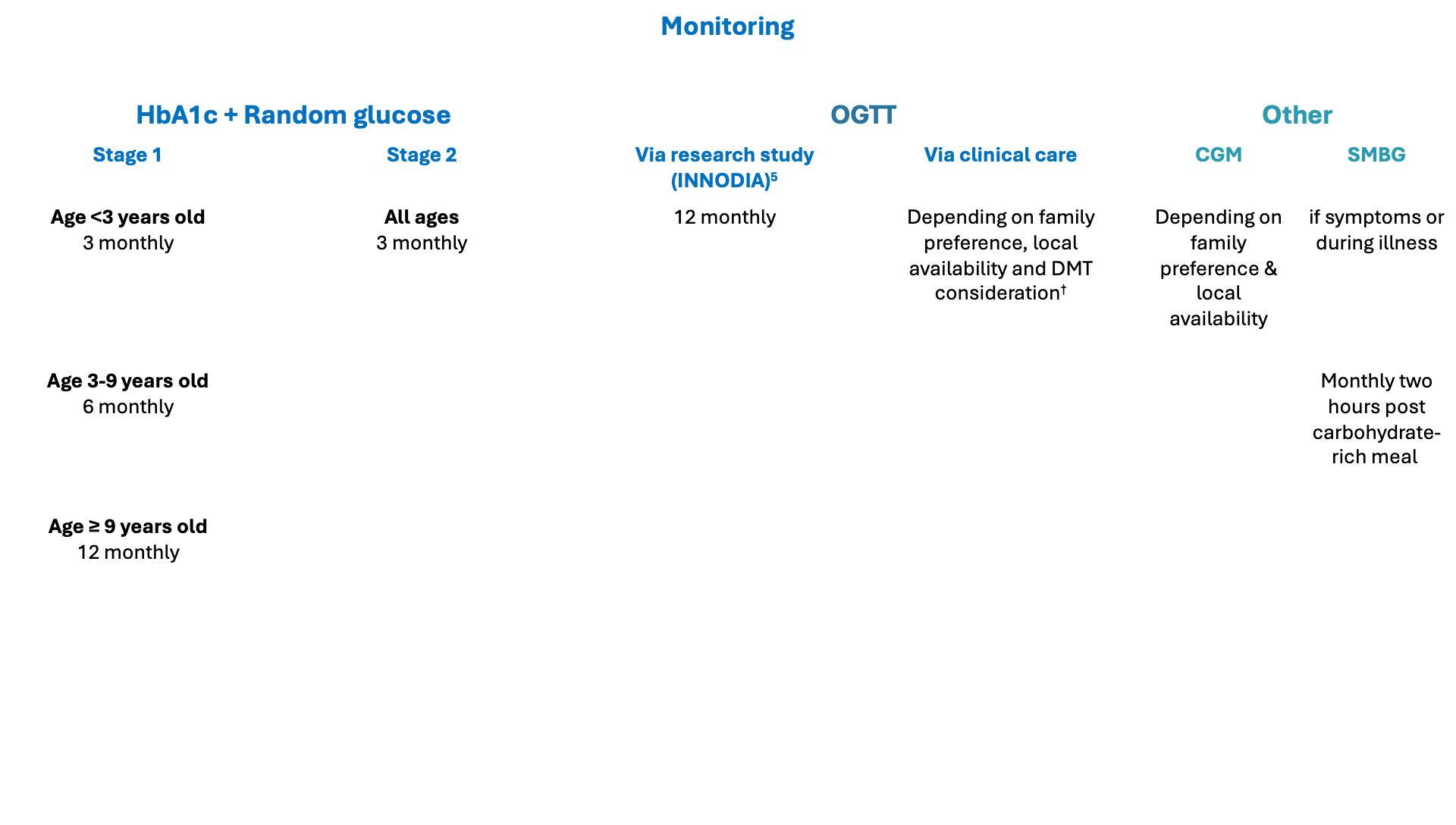
**

**Supplementary figure 1** **Legend:** The surveillance approach was developed at service implementation and refined as international and national consensus guidance became available.^1,3,4^
† OGTT performed in clinical care when stage progression was suspected (e.g. HbA1c increase ≥10% from baseline), when disease-modifying therapy (DMT) eligibility was being considered, or based on family preference and local service availability.
**Abbreviations:** HbA1c = glycated haemoglobin; OGTT = oral glucose tolerance test; INNODIA = **Inn**ovative approach towards understanding and arresting type 1 diabetes; CGM = continuous glucose monitoring; SMBG = self-monitored blood glucose; DMT = disease-modifying therapy.

**Supplementary Figure 2** Family Survey Responses Before and After Clinic Attendance

**Supplementary Figure 2 Legend.** Bar graph illustrating median Likert points for type 1 diabetes (T1D) Empowerment and T1D Knowledge before and after attending the clinic. Participants rated their perceived empowerment and knowledge related to early-stage type 1 diabetes on a scale from 1 (lowest) to 10 (highest). The graph displays median values with interquartile ranges, illustrating increases in both empowerment and knowledge following participation in the clinic. Abbreviations: T1D = type 1 diabetes.

**References**

1. Haller MJ, Bell KJ, Besser REJ, et al. ISPAD Clinical Practice Consensus Guidelines 2024: screening, staging, and strategies to preserve beta-cell function in children and adolescents with type 1 diabetes. Horm Res Paediatr. 2024;97(6):529-545. doi:10.1159/000543035
2. American Diabetes Association Professional Practice Committee. 2. Diagnosis and classification of diabetes: Standards of Care in Diabetes—2024. Diabetes Care. 2024;47(Suppl 1):S20-S42. doi:10.2337/dc24-S002
3. Phillip M, Achenbach P, Addala A, et al. Consensus guidance for monitoring individuals with islet autoantibody–positive pre–stage 3 type 1 diabetes. *Diabetes Care*. Published online June 24, 2024. doi:10.2337/dci24-004
4. Besser REJ, Campbell F, Damazer K, et al. UK best practice recommendations for children and young people <18 years with pre–stage 3 type 1 diabetes: a British Society for Paediatric Endocrinology and Diabetes (BSPED) statement. *Diabet Med*. 2025;42(11):e70117
5. Dunger DB, Bruggraber SFA, Mander AP, et al. INNODIA Master Protocol for the evaluation of investigational medicinal products in children, adolescents and adults with newly diagnosed type 1 diabetes. *Trials*. 2022;23:414. doi:10.1186/s13063-022-06259-z
